# Nutraceutical potential of prekese (*Tetrapleura tetraptera*) fruit extract: synergizing growth performance, oxidative stability, and economic viability in Nile tilapia (*Oreochromis niloticus*)

**DOI:** 10.3389/fphys.2026.1869905

**Published:** 2026-08-12

**Authors:** Samuel B. Umma, Yetunde E. Agbeja, Raphael Z. Agu, Bilal A. Paray, Eijaz A. Bhat, Robert M. Iorhiin

**Affiliations:** 1 Department of Fisheries and Aquaculture, Federal University Wukari, Wukari, Taraba State, Nigeria; 2 Department of Aquaculture and Fisheries Management, University of Ibadan, Ibadan, Nigeria; 3 Department of Zoology, King Saud University, Riyadh, Saudi Arabia; 4 Molecular Physiology of Prokaryotes, Institute of Biology II, University of Freiburg, Freiburg, Germany; 5 Department of Fisheries and Aquaculture, University of Calabar, Calabar, Nigeria

**Keywords:** fatty acid profile, fingerlings, growth performance, immunomodulation, nutraceuticals

## Abstract

This study evaluated the effects of dietary Prekese (*Tetrapleura tetraptera*) fruit extract (PFE) on the growth, haemato-biochemical profile, antioxidant status, and economic efficiency of Nile tilapia (*Oreochromis niloticus*) fingerlings. Five isonitrogenous (35% crude protein) and isolipidic diets were formulated with graded PFE levels: 0.0 (control), 5.0, 10.0, 15.0, and 20.0 g/kg. Fish (5.20 ± 0.05 g) were fed twice daily to satiety for 60 days. Gas chromatography- mass spectral analysis of PFE identified 29 compounds, primarily linoleic (20.46%), myristic (14.53%), and oleic (12.49%) acids. Dietary PFE significantly enhanced growth in a dose-dependent manner (P< 0.05). The 20.0 g/kg inclusion yielded a higher final body weight (35.04 g) and weight gain (571.97%), a 121.9% increase over the control. Polynomial contrasts identified 20.0 g/kg as the upper limit for growth and feed efficiency in this study. Haematologically, PFE increased red and white blood cell counts, suggesting improved hematopoietic and immune function. Serum biochemistry showed elevations in aspartate aminotransferase, alanine aminotransferase, and alkaline phosphate at 15.0 – 20.0 g/kg PFE, though within physiological ranges. Importantly, PFE supplementation significantly reduced oxidative stress, evidenced by decreased superoxide dismutase, catalase, and malondialdehyde levels (P< 0.05), indicating enhanced systemic antioxidant capacity. Economic analysis revealed that despite a 96.73% increase in feed cost, 20.0 g/kg PFE increased net profit per fish by 136.8% and significantly improved the benefit-cost ratio. In conclusion, PFE is a viable nutraceutical for Nile tilapia. At a higher inclusion of 20.0 g/kg, PFE boosts growth, mitigates oxidative stress, and maximizes economic returns in sustainable aquaculture.

## 1 Introduction

Global food systems face unprecedented pressure to reconcile rising demands with sustainable production models, aligning with the United Nations Sustainable Development Goal 2 (SDG-2) to eliminate hunger and establish global nutritional security (United Nations Economic and Social Council, 2024). Sustainable intensification of animal agriculture can mitigate the expanding global protein deficit while meeting these production targets. The global demand for animal-derived protein will double its current baseline between 2000 and 2050, driven by rapid population growth and urbanizing dietary shifts in emerging economies (Henchion et al., 2021). Aquaculture constitutes the world’s fastest-growing food sector and currently supplies roughly 15.3% of global animal protein intake (Boyd et al., 2022). Nevertheless, commercial scalability remains constrained by escalating production costs and a volatile supply of conventional, high-quality feed ingredients. The long-term viability of modern aquaculture thus hinges on precise nutritional interventions and the formulation of cost-effective, highly bioavailable diets (Girão et al., 2026).

Nile tilapia remains central to this blue revolution, serving as the second most extensively cultivated finfish group globally (FAO, 2024). Since the expansion of commercial domestication in the mid-20th century, tilapias have achieved global production prominence due to their high reproductive plasticity, environmental resilience, and efficient utilization of varied dietary protein regimes (McConnell, 2021). Because this species underpins small and large scale economies across developing regions, particularly within West Africa, optimizing production through targeted nutritional strategies directly impacts regional food security and rural poverty alleviation initiatives (Chan et al., 2019).

However, high-density intensive culture systems frequently expose fish to chronic physiological stressors, which impair somatotropic performance and compromise immunocompetence (Martos-Sitcha et al., 2020). This challenge has accelerated research into functional feeds enriched with plant-derived phytobiotics. Phytogenic extracts present a viable, eco-friendly alternative to synthetic antibiotics and therapeutic chemicals, serving as natural growth promoters and immunomodulators (Umma et al., 2025; Okon et al., 2025). A promising, yet underutilized botanical candidate is *Tetrapleura tetraptera* (locally termed Prekese). This deciduous tree, indigenous to West Africa, produces fruits highly enriched with bioactive secondary metabolites notably saponins, flavonoids, and tannins which exhibit potent therapeutic and physiological properties (Mensah et al., 2024).

Although the pharmacological profile of prekese is well-characterized in mammalian models, its role in fish nutritional physiology remains poorly understood. Specifically, empirical data detailing how dietary PFE alters the bioenergetics, blood profile, and microeconomic profitability of tilapia production are lacking. This lack of studies creates a practical risk for the industry, and without clear physiological thresholds, feed formulators are essentially working in the dark, which can easily lead to accidental anti-nutritional toxicity, compromised fish health, and wasted capital. Consequently, the absence of standardized inclusion frameworks leaves tilapia producers vulnerable to unforeseen metabolic stress and severe financial losses, stalling efforts to make regional aquaculture truly sustainable.

## 2 Materials and methods

### 2.1 Extraction and phytochemical profiling of prekese fruit extract

Mature fruits of *Tetrapleura tetraptera* (prekese) were sourced locally, authenticated by a taxonomist at the University Herbarium, and processed by rinsing and sun-drying to a constant weight. The dried fruits were pulverized and subjected to thermal liquid-state maceration in distilled water (1:10, w/v ratio) using a laboratory water bath (VWR WB10, OH, USA) at 60 °C for 48 hours. The resulting liquid extract was filtered through a 120-micron nylon mesh and dehydrated in a vacuum drying oven (Zenith Lab, China) at 40 °C under reduced pressure for 72 hours, yielding a concentrated dry PFE powder (extraction yield: 14.2%, w/w). Qualitative screening for alkaloids, saponins, tannins, and oxalates was conducted, followed by quantitative characterization via Gas Chromatography-Mass Spectrometry (GC-MS) for bioactive compounds and Atomic Absorption Spectroscopy (AAS) for mineral quantification (AOAC, 2005; Umma et al., 2023, 2025).

For GC-MS analysis, a gas chromatograph (Agilent 7890B) coupled with a mass spectrometer (Agilent 5977B) equipped with an HP-5MS capillary column (30 m × 0.25 mm ID × 0.25 μm film thickness) was utilized. Helium served as the carrier gas at a constant flow rate of 1.0 mL min^-1^. The injector temperature was maintained at 250 °C with a split ratio of 10:1. The oven temperature program began at 60 °C (held for 2 min), ramped to 200 °C at 10 °C min^-1^, and finally increased to 280 °C at 5 °C min^-1^ (held for 10 min). Mass spectra were acquired in electron ionization (EI) mode at 70 eV with a scan range of m/z 50 – 550. Compounds were identified by comparing their mass spectra and retention indices with the National Institute of Standards and Technology (NIST17) spectral database.

### 2.2 Experimental fish, diet formulation, and culture management

Nile tilapia fingerlings (Initial weight: 5.20 ± 0.05 g) were acclimated for 14 days and fed a commercial starter diet (Aller Aqua, Denmark). Five isonitrogenous and isocaloric experimental diets (35% Crude Protein) were formulated using Pearsons square method. Pulverized fish-feed ingredients were fortified with PFE at 0.0, 5.0, 10.0, 15.0 20.0 g/kg (**Table 1**), and thoroughly mixed with warm water (100 mL per 1000 g dry matter [DM]) to form dough and were pelleted using a 2 mm pellet size dry pelleting machine (Model: LM50, Henan Lima Machinery Manufacture Co. LTD., Zhengzhou, China). The pelleted feeds were produced in batches of 1 kg fortnightly and stored at 4 °C.

**Table 1:** Ingredients and proximate composition of PFE diets (g/kg DM).

Ingredients	0.0 g PFE	5.0 g PFE	10.0 g PFE	15.0 g PFE	20.0 g PFE
Fish meal (70% CP)	225	225	225	225	225
Soybean meal (44% CP)	225	225	225	225	225
Yellow Maize (8% CP)	460	455	450	445	440
PFE (g/kg)	0	5	10	15	20
*Vitamin and Mineral Premix	5	5	5	5	5
Binder (Starch)	40	40	40	40	40
Vegetable Oil	45	45	45	45	45
Total	1000	1000	1000	1000	1000
Proximate composition (Analysed)					
Crude Protein (%)	35.1	35	35.2	34.9	35.1
Crude Fat (%)	6.5	6.6	6.4	6.5	6.4
Crude Fibre (%)	4.2	4.1	4.3	4.2	4.2
Ash (%)	8.5	8.7	8.4	8.6	8.5
Moisture	8.2	8.4	7.9	8.1	8.1
NFE Estimate	37.5	37.2	37.8	37.7	37.7

*Vitamin and Mineral premix: Calcium pantothenate: 60 mg, Choline chloride: 500 mg, Inositol: 50 mg, Manganese: 30 mg, Iron: 35 mg, Zinc: 45 mg, Copper: 3 mg, Iodine: 5 mg, Cobalt: 2 mg, Selenium: 0.15 mg, Anti-oxidant: 80 mg. vitamin A: 22;000 I.U; vitamin D3: 5000 I.U; vitamin E: 300 mg; vitamin K3: 10 mg; vitamin B1: 20 mg; vitamin B2: 25 mg; vitamin C: 300 mg; niacin: 120 mg; vitamin B6: 10 mg; vitamin B12: 0.05 mg; folic acid: 5 mg; biotin: 1 mg.

A total of 150 fish were randomly distributed into 15 tanks/experimental units (50 L capacity) at a density of 10 fish per tank, representing three replicates per group (n = 3). Fish in each experimental group were fed to apparent satiation twice daily at 09:00 and 16:00 h for a period of 60 days. Total feed consumption was determined at the end of the trial by subtracting the weight of unconsumed, recovered feed from the initial amount supplied. Water temperature was monitored daily at 09:00 and 16:00 h using a digital multiparameter probe (HI98129, Hanna Instruments, Woonsocket, RI, USA as follows: Temperature (28.0 − 28.5 °C), Dissolved Oxygen (6.0 7 − 6.38 mg/L), and pH (6.83 − 6.93). At the trial’s conclusion, growth performance was evaluated using standard indices (Equations 1–3):

(1) Weight gain (WG, g)=Final weight (g)−Initial weight (g)

(2) $$\text{Specific Growth Rate}\;\left(\text{SGR, }\%\;day^{-1}\right)=\frac{\text{Ln Final weight}-\text{Ln Initial weight}}{\text{Duration of culture}\;(\text{days})}\times 100$$

(3) $$\text{Feed Conversion Ratio}\;\left(\text{FCR}\right)=\frac{\text{Fish feed intake}\left(\text{g}\right)}{\text{Fish weight gain}\left(\text{g}\right)}$$

### 2.3 Haematological and serum biochemical analysis

Following a 24-hour fast, fish were anesthetized using buffered MS-222 (30 mg/L, concentration). Blood samples were collected from the caudal vein using one cubic centimetre heparinized syringes (BD Preset™, Becton, Dickinson and Company, Franklin Lakes, NJ, USA) in replicates (n = 3 fish per replicate tank; n = 9 fish per treatment). Samples were divided into EDTA-treated tubes for haematological parameters [Packed cell volume (PCV), Haemoglobin (Hb), Red blood cell (RBC), white blood cell (WBC)] and non-heparinized tubes for serum biochemistry (Total Protein, Albumin, Glucose), using the protocol described by Hoque and Das (2025), as follows:

#### 2.3.1 Haematological analysis

Haematological parameters were determined immediately following blood sampling. Packed cell volume (PCV, %) was evaluated using the microhaematocrit method; non-heparinized capillary tubes were filled to approximately 75% capacity with anticoagulated whole blood, sealed with non-absorbent clay, and centrifuged at 10,000 × g for 5 minutes before reading on a microhaematocrit scale. Haemoglobin concentration Hb, g/dL was determined colorimetrically via the cyanmethaemoglobin method using Drabkin’s reagent. Briefly, 20 μL of whole blood was mixed with 5.0 mL of Drabkin’s reagent, incubated at room temperature for 10 min, and the absorbance was measured at 540 nm against a reagent blank using a spectrophotometer.

Red blood cell (RBC) and white blood cell (WBC) counts (μ/L) were determined manually using an improved Neubauer haemocytometer. Whole blood (20 μL) was diluted in Natt-Herrick’s solution (4.0 mL) to achieve a 1:200 dilution ratio. The mixture was charged into the counting chamber and allowed to settle for 3 minutes in a humidified environment. Red blood cells were quantified within the five central small squares under a light microscope at 400 × magnification, whereas white blood cells were counted across the four large corner squares at 100 × magnification. Final counts were computed incorporating the specific dilution and chamber volume correction factors.

#### 2.3.2 Serum biochemical analysis

For serum separation, collected blood samples (without anticoagulant) were allowed to clot at room temperature and subsequently centrifuged at 3,000 × g at 4 °C for 10 minutes. The resulting clear serum was aliquoted and stored at -20 °C ready for analysis.

Serum biochemical profiles were determined colorimetrically using standard diagnostic protocols. Total protein concentration (g/dL) was measured via the Biuret method at 540 nm following incubation at 37 °C for 10 minutes. Serum albumin (g/dL) was quantified using the Bromocresol Green (BCG) method, measuring absorbance at 628 nm after a 5-min room-temperature incubation. Serum glucose (mg dL) was determined using the enzymatic Glucose Oxidase–Peroxidase (GOD-POD) method at 505 nm after incubation at 37 °C for 10 minutes. All parameters were calculated relative to the optical densities of their respective standard solutions according to the general formula:

$\text{Concentration of Sample}=\frac{\text{Absorbance of Sample}}{\text{Absorbance of Standard }}\times \text{Concentration of Standard}$

### 2.4 Economic efficiency analysis

An economic cost-benefit analysis was conducted at the conclusion of the 60-day feeding trial to evaluate the financial feasibility of dietary PFE supplementation in Nile tilapia feeds. Local market prices (expressed in NGN currency at the time of the trial: Base diet ingredient cost = 1,275 per kg; PFE raw processing cost = 3,750 per kg; Fish market value = 4,800 per kg) were used to compute indices following standardized formulas (Equations 4–8) (Lozano et al., 2007; Yakubu et al., 2014):

(4) $$\text{Incidence of cost}=\frac{\text{Cost of feed per kg}}{\text{Weight gain}\left(\text{kg}\right)}$$

This parameter quantifies the direct feed cost required to produce a kilogram of fish biomass, serving as a critical indicator of relative production costs.

(5) $$\text{Profit Index}\;(\text{PI})=\frac{\text{Value of fish produced per Kg}}{\text{Cost of feed}}$$

The profit index serves as a specific measure of financial return generated relative to feed investment, where a higher PI signifies a more cost-effective utilization of dietary nutrients into marketable biomass.

(6) $$\text{Benefit-Cost}\;\text{Ratio}\;\left(\text{BCR}\right)=\frac{\text{Total revenue}}{\text{Total Expenditure}}$$

A BCR greater than 1.0 establishes the baseline for commercial profitability of the dietary treatments.

(7) $$\text{Economic feed conversion ratio}\;(\text{EFCR})=\frac{\text{Quantity of feed consumed}\times \text{Cost per Kg }}{\text{Weight gain}\left(\text{kg}\right)}$$

The lower the EFCR, the better, similar to the biological FCR. A lower EFCR value indicates superior economic efficiency, which means that a farmer will spend less on feed to generate a higher financial return in fish flesh.

(8) $$\text{Net profit margin}=\frac{\text{Total Revenue}-\text{Total Cost}}{\text{Total Revenue}}\times 100$$

This metric reflects the structural percentage of revenue retained as profit after accounting for all operational feed expenses.

All data were tested for normality using the Anderson–Darling test and for homogeneity of variance using Levene’s test. The experimental tank served as the primary experimental unit (n = 3). Data were subjected to a One-Way Analysis of Variance (ANOVA) at a significance threshold of α = 0.05. Where significant differences were detected, Fisher’s Least Significant Difference (LSD) *post-hoc* test was applied for pairwise mean comparisons. Furthermore, orthogonal polynomial contrasts (linear and quadratic) were calculated to evaluate response trends associated with graded PFE inclusion levels on fish growth and feed efficiency parameters. All statistical analyses were executed using Minitab^®^ (Version 19, Minitab LLC, State College, PA, USA).

## 3 Results

### 3.1 Phytochemical and bioactive characterization of PFE

**Figure 1:**
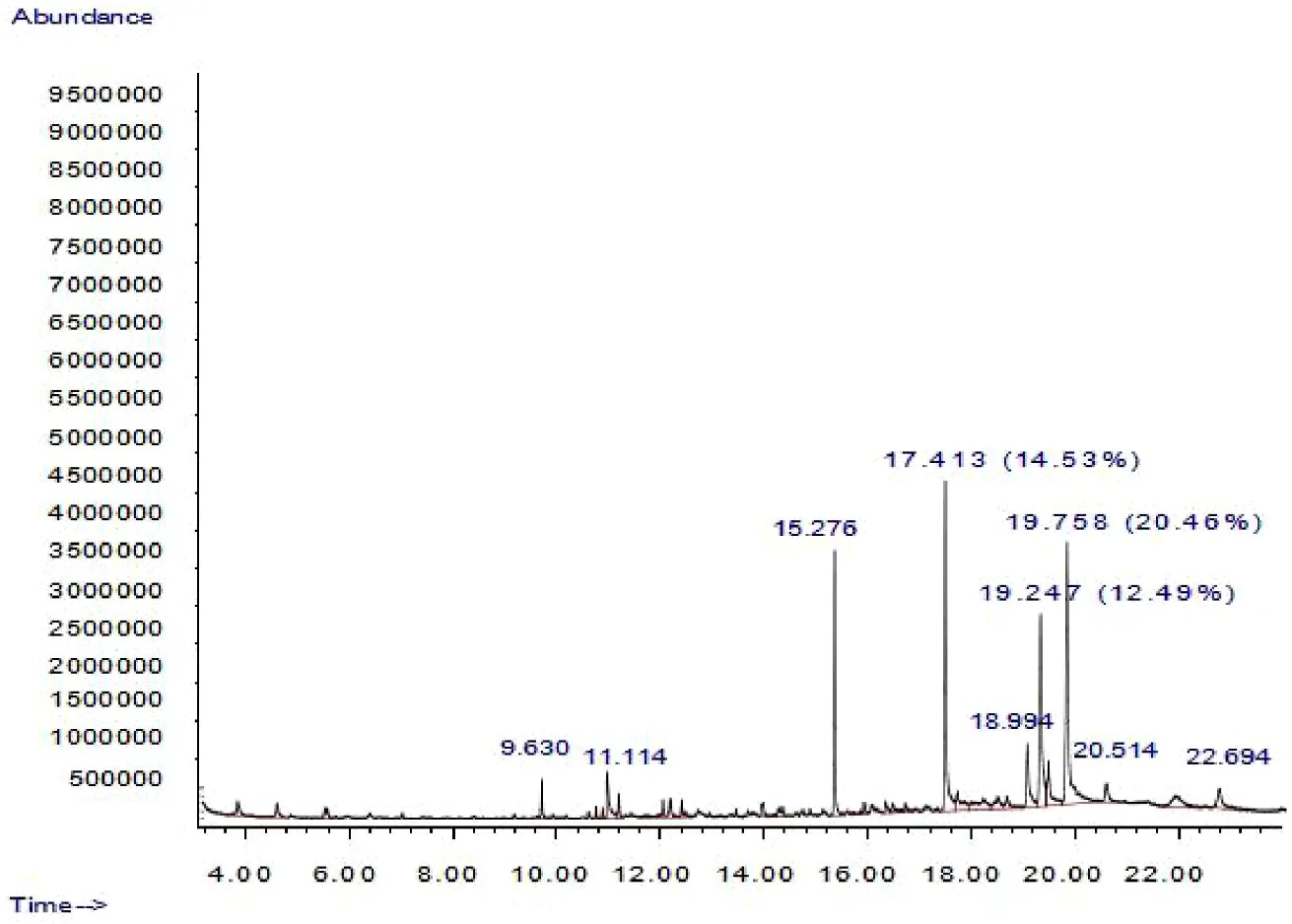
Total ion chromatogram of PFE indicating the distinguishable peaks (Abundance) against the retention time.

**Figure 2:**
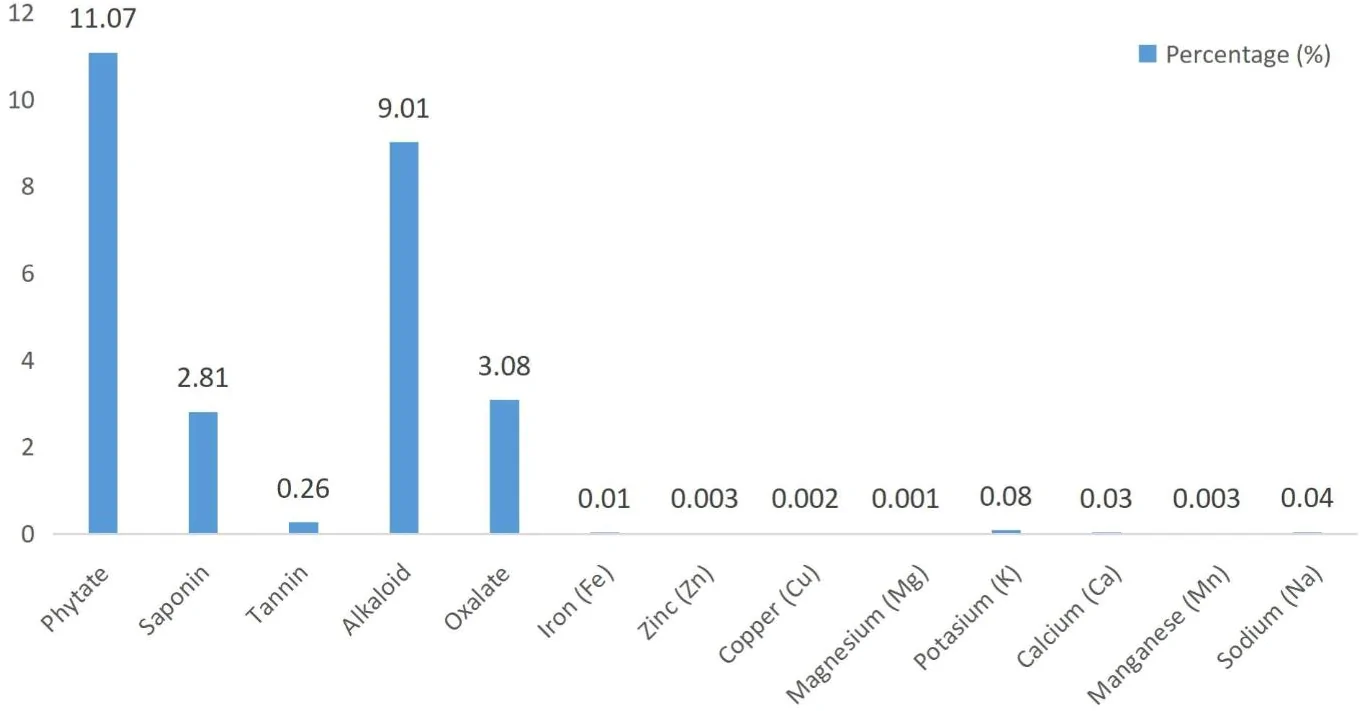
Percentage of phytochemicals and mineral content in PFE sample.

The Total Ion Chromatogram (TIC) of the prekese fruit extract (PFE) identified 29 distinct bioactive volatiles (**Figure 1**). Long-chain fatty acids (LCFAs) dominated this chemical profile, making up nearly half of the total volatile landscape (47.48%). Structurally, 9,12-octadecadienoic acid (Linoleic Acid; 20.46%), tetradecanoic acid (Myristic Acid; 14.53%), and oleic acid (12.49%) emerged as the primary constituents, all matching established spectral baselines with high confidence (Quality ≥ 91). Quantitative secondary metabolite and mineral profiling showed a diverse chemical background where phytate (11.07%) and tannins (0.26%) were the principal anti-nutritional factors, while potassium (0.08%) was the most abundant mineral (**Figure 2**).

### 3.2 Growth performance and feed utilization

The inclusion of PFE in the diets of Nile tilapia caused significant, dose-dependent improvement in all growth metrics (**Table 2**; P< 0.05). The FBW rose linearly up to the 20.0 g/kg inclusion level, outgrowing the control group (0.0 g/kg) by 50.5%. WG reflected this trend, climbing from 200.29% in the control fish to 571.97% in the 20.0 g/kg treatment group. The SGR likewise showed a significant upward progression, rising from 2.09%day^-^¹ in the control to 2.91%day^-^¹ in the 20.0 g/kg PFE group.

**Table 2:** Growth and nutrient utilization of Nile tilapia fed dietary PFE.

Parameters	0.0 g/kg	5.0 g/kg	10.0 g/kg	15.0 g/kg	20.0 g/kg	Pooled St.Dev	Linear contrast	Quadratic contrast
Initial wt (g)	5.26 ± 0.02^a^	5.140 ± 0.01^a^	5.19 ± 0.09^a^	5.16 ± 0.0^a^	5.22 ± 0.0^a^	0.05	0.48	0.07
FBW (g)	15.79 ± 0.08^a^	17.87 ± 0.19^b^	26.79 ± 0.12^c^	31.53 ± 0.11^d^	35.04 ± 0.08^e^	7.91	< 0.01	0.01
WG (%)	200.29 ± 2.74^e^	247.51 ± 4.70^d^	416.87 ± 12.09^c^	511.31 ± 2.09^b^	571.97 ± 1.55^a^	6.05	< 0.01	0.03
TFI	16.00 ± 0.07^e^	18.28 ± 025^d^	27.80 ± 021^c^	31.73 ± 0.25^d^	33.80 ± 0.21^e^	2.37	< 0.01	< 0.01
FCR	1.52 ± 0.02^a^	1.42 ± 0.02^b^	1.29 ± 0.01^c^	1.21 ± 0.01^d^	1.10 ± 0.02^e^	0.02	< 0.01	0.50
SGR (% day^-^¹)	2.09 ± 0.01^e^	2.26 ± 0.02^d^	2.65 ± 0.04^c^	2.82 ± 0.0^b^	2.91 ± 0.0^a^	0.02	< 0.01	< 0.01
FER	0.66 ± 0.01^e^	0.70 ± 0.01^d^	0.78 ± 0.01^c^	0.83 ± 0.0^b^	0.91 ± 0.01^a^	0.01	< 0.01	0.06
PER	0.67 ± 0.0^e^	0.71 ± 0.0^d^	0.81 ± 0.01^c^	0.84 ± 0.0^b^	0.85 ± 0.0^a^	0.0	< 0.01	< 0.01

Means that do not share a letter across the row are significantly different (P< 0.05).

Notably, TFI rose significantly with higher PFE levels, suggesting a phagostimulatory effect of the extract. This increased consumption occurred alongside a significant drop in the feed conversion ratio (FCR), which fell from 1.52 to 1.10 (P< 0.05). Both feed efficiency (FER) and protein efficiency (PER) showed strong linear and quadratic responses.

**Figure 3:**
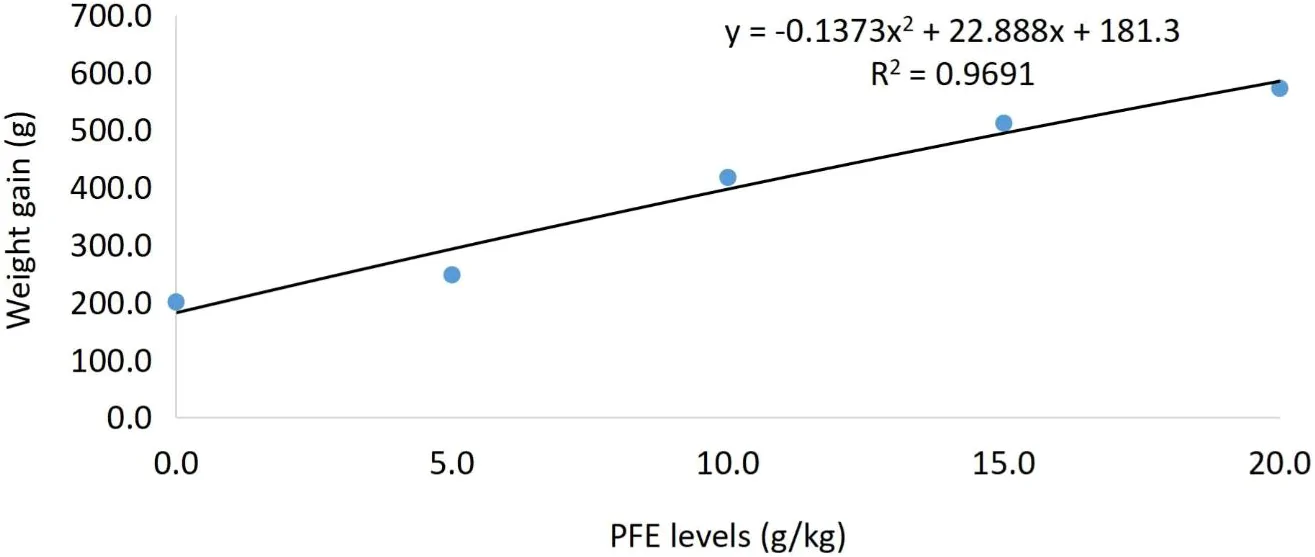
The relationship between weight gain (g) and PFE levels.

**Figure 4:**
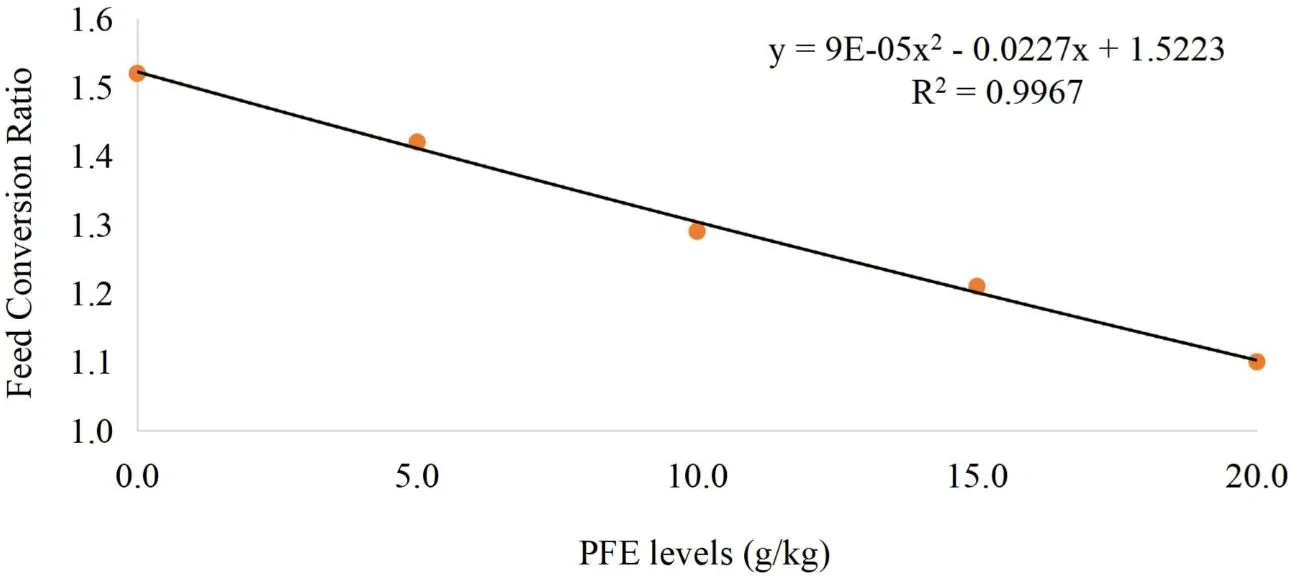
The relationship between feed conversion ratio and PFE levels.

**Figure 5:**
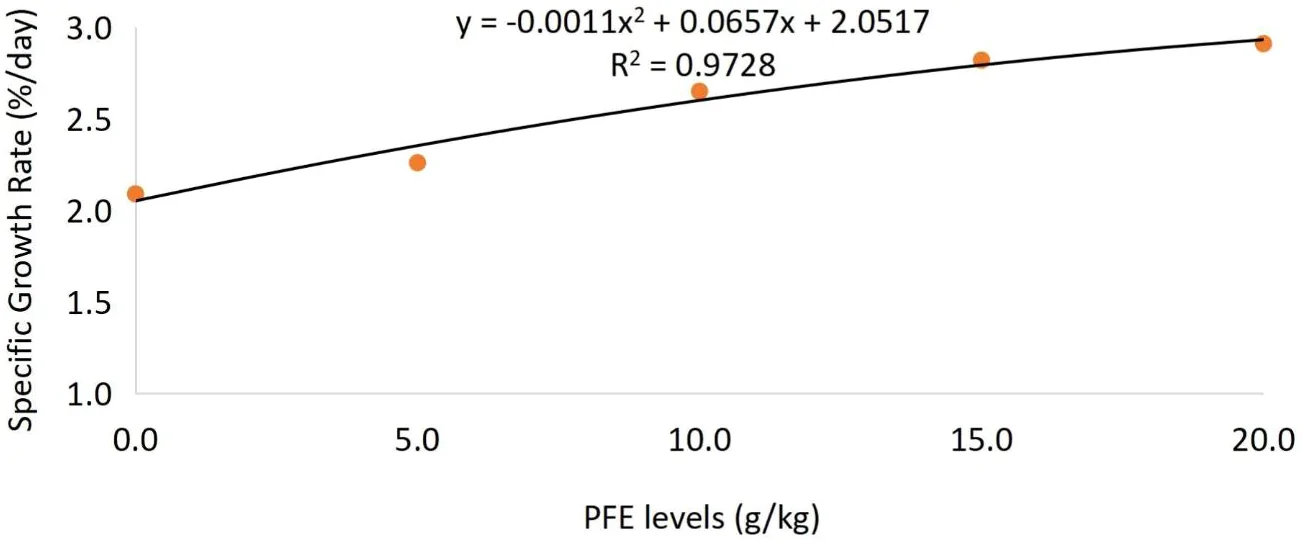
The relationship between specific growth rate and PFE levels.

Second-order polynomial contrasts analysis was employed to model the relationship between dietary PFE levels (x) and key performance indices (y), (**Figures 3**–**5**). The resulting equations were as follows:

$\text{WG: y}=181.3+22.888\text{x}-0.1373{\text{x}}^{2}\;\left({\text{R}}^{2}=0.9691\right)$ $\text{SGR: y}=2.0517+0.0657\text{x}-0.0011{\text{x}}^{2}\;\left({\text{R}}^{2}=0.9728\right)$ $\text{FCR: y}=1.5223-0.0227\text{x}+9\text{E}-05{\text{x}}^{2}\;\left({\text{R}}^{2}=0.9967\right)$The WG model confirmed a highly reliable quadratic fit (R^2^ = 0.9691). The positive linear coefficient (22.888) underlines the strong initial growth-promoting effect of the extract, while the negative quadratic component (-0.1373) shows a typical late-stage growth saturation trend. Although solving for the vertex (x = −b/2a), it indicates a theoretical maximum tolerance threshold of 83.35 g/kg, proving that the safe biological limits of Nile tilapia were never threatened within our 0.0 to 20.0 g/kg experimental range.

The FCR model (R^2^ = 0.9967) reveals a near-perfect mathematical relationship, driven by a linear coefficient of -0.0227, meaning that every gram of PFE added per kilogram of feed drops the FCR by roughly 0.02 units. The SGR model (R^2^ = 0.9728) established a baseline control intercept of 2.05% day^-1^, with its negative quadratic term (-0.011) placing the theoretical biological peak at 29.86 g/kg, which suggests that absolute maximum efficiency sits just beyond our highest tested dose.

### 3.3 Haematological modulation

Dietary PFE treatments altered the blood profiles of Nile tilapia, creating distinct, cell-specific changes (**Table 3**; P< 0.05). The WBC counts and haematocrit percentages rose linearly (P< 0.05) as PFE inclusion climbed from 0.0 to 20.0 g/kg. The PCV and Hb concentrations remained steady across all treatments, though a minor, non-significant peak was recorded in the 20.0 g/kg group (8.15 g/dL).

**Table 3:** Haematological profile of Nile tilapia fed dietary PFE.

Parameters	0.0 g/kg	5.0 g/kg	10.0 g/kg	15.0 g/kg	20.0 g/kg	Pooled Stdev	Linear contrast	Quadratic contrast
PCV (%)	26.50 ± 0.71^ab^	27.50 ± 0.71^a^	26.00 ± 0.0^b^	26.50 ± 0.71^ab^	26.00 ± 0.0^b^	0.55	0.16	0.18
Hb (g/dL)	7.60 ± 0.57^ab^	7.55 ± 0.07^ab^	7.05 ± 0.71^b^	7.20 ± 0.14^b^	8.15 ± 0.21^a^	0.2 8	0.29	0.02
RBC (10^12^/L)	2.00 ± 0.0^c^	2.25 ± 0.07^a^	2.00 ± 0.0^b^	2.35 ± 0.07^a^	1.75 ± 0.07^c^	0.05	0.02	< 0.01
WBC (10^9^/L)	11.15 ± 0.07^c^	11.35 ± 0.07^c^	12.10 ± 0.14^b^	12.55 ± 0.07^a^	12.6 ± 0.14^a^	0.10	< 0.01	0.08
Haematocrit (%)	61.00 ± 1.41^c^	67.00 ± 1.41^b^	66.50 ± 0.70^b^	68.00 ± 0.0^ab^	70.0 ± 0.0^a^	0.95	< 0.01	0.06
Lymphocyte (%)	64.00 ± 1.41^b^	79.00 ± 1.41^a^	52.50 ± 3.54^c^	30.50 ± 0.71^d^	25.50 ± 0.71^e^	1.87	< 0.01	< 0.01
Monocytes (%)	0.0 ± 0.0^e^	0.0 ± 0.0 d	1.00 ± 0.0^b^	1.00 ± 0.0^a^	0.0 ± 0.0^c^	0.0	< 0.01	< 0.01
Eosinocytes (%)	0.0 ± 0.0^c^	2.00 ± 0.0^b^	2.00 ± 0.0^b^	0.0 ± 0.0^c^	3.50 ± 0.71^a^	0.32	< 0.01	0.29

Means that do not share a letter across the rows are significantly different (P< 0.05).

The differential leukocyte counts shifted drastically across the dietary treatments. Circulating lymphocytes fell from a maximum of 79.00% in the 5.0 g/kg group to a low of 25.50% in the 20.0 g/kg group. At the same time, eosinocytes which were barely visible in control fish became increasingly common at higher PFE doses. Monocytes were mostly undetectable across the board, appearing only within the mid-range 10.0 and 15.0 g/kg PFE groups.

### 3.4 Serum biochemical markers and antioxidant status

The serum biochemical profile of Nile tilapia exhibited significant sensitivity to dietary PFE inclusion, characterized by both linear and quadratic modulations (**Table 4**). A significant increase (P< 0.05) was observed in the activities of ALP, AST, and ALT as PFE levels escalated. Specifically, AST values rose from 56.50 IU/L in the control group to a peak of 70.50 IU/L in the 20.0 g/kg group. Similarly, GGT activity was significantly elevated, reaching a higher response limit in the 15.0 g/kg groups.

**Table 4:** Serum biochemical markers of Nile tilapia fed dietary PFE.

Parameters	0.0 g/kg	5.0 g/kg	10.0 g/kg	15.0 g/kg	20.0 g/kg	Pooled St.Dev	Linear contrast	Quadratic contrast
AST (µ/L)	56.50 ± 0.71^c^	67.50 ± 0.71^b^	68.25 ± 0.35^b^	68.50 ± 0.71^b^	70.50 ± 0.71^a^	0.65	< 0.01	< 0.01
ALT (µ/L)	18.75 ± 0.71^b^	18.50 ± 0.71^b^	18.75 ± 0.35^b^	22.50 ± 0.71^a^	23.50 ± 0.71^a^	0.65	< 0.01	< 0.01
ALP (µL)	66.44 ± 0.62^c^	60.34 ± 0.47^e^	62.50 ± 0.71^d^	69.84 ± 0.23^b^	77.33 ± 0.46^a^	0.52	< 0.01	< 0.01
UREA (mg/dL)	36.78 ± 1.09^ab^	36.24 ± 1.05^b^	36.25 ± 0.35^b^	38.55 ± 0.77^a^	38.23 ± 0.32^ab^	0.78	0.02	0.36
Creatinine (mg/dL)	0.58 ± 0.11^a^	0.52 ± 0.02^a^	0.40 ± 0.0^b^	0.40 ± 0.0^b^	0.40 ± 0.01^b^	0.05	0.14	< 0.01
GGT (µ/L)	42.95 ± 0.08^bc^	40.51 ± 0.72^d^	42.00 ± 0.0^c^	44.95 ± 0.09^a^	44.38 ± 0.54^b^	0.41	< 0.01	0.03
MDA (mmol/mg protein)	1.79 ± 0.14^b^	2.69 ± 0.26^a^	1.80 ± 0.14^b^	1.27 ± 0.05^c^	1.74 ± 0.05^b^	0.15	< 0.01	< 0.01
GSH (µg/mL)	3.68 ± 0.81^b^	3.25 ± 0.71^c^	3.25 ± 0.07^c^	3.17 ± 0.32^a^	3.68 ± 0.04^b^	0.50	< 0.01	0.77
CAT (µmol/mg protein)	28.99 ± 1.40^a^	24.17 ± 0.24^b^	23.25 ± 0.35^bc^	25.39 ± 0.6^c^	23.12 ± 0.17^c^	0.70	< 0.01	0.01
SOD (µmol)	36.27 ± 0.38^a^	34.49 ± 0.70^c^	35.0 ± 0.71^bc^	35.88 ± 0.17^ab^	35.88 ± 0.17^ab^	0.49	0.78	0.02

Means that do not share a letter across the rows are significantly different (P< 0.05).

Renal biomarkers and nitrogenous products were heavily influenced by the diets. Serum urea concentrations increased linearly, peaking in the highest inclusion group, while creatinine values dropped to their lowest levels within the 10.0 to 20.0 g/kg treatments.

In terms of oxidative stress and antioxidant status, MDA levels dropped significantly in the 15.0 g/kg group compared to the control. While reduced GSH levels remained statistically uniform across all treatments (P > 0.05), the internal enzymatic defences moved downward.CAT activity fell significantly from the control to the 20.0 g/kg group, and SOD activity remained higher in the unsupplemented control fish. This pattern suggests a complex interaction where the exogenous bioactive compounds in PFE may be augmenting the systemic antioxidant capacity, thereby reducing the demand for endogenous enzymatic responses.

### 3.5 Economic efficiency analysis of Nile tilapia fed dietary PFE

The bio-economic evaluation clearly demonstrated that the improved growth performance of the Nile tilapia generated a clear commercial advantage across the treatment groups (**Table 5**; P< 0.05). The addition of PFE introduced a distinct quadratic increase in the unit cost of feed, peaking at a 96.7% increase for the 20.0 g/kg group compared to the control (0.0 g/kg). However, this cost was significantly neutralized by a major improvement in feed efficiency, as the EFCR dropped from 3.13 in the control down to 1.99 in the 20.0 g/kg treatment group. This demonstrates that PFE functions as a metabolic catalyst that extracts more value per unit of feed.

**Table 5:** Economic performance of Nile tilapia fed dietary PFE.

Parameters	0.0 g/kg	5.0 g/kg	10.0 g/kg	15.0 g/kg	20.0 g/kg	Pooled St.Dev	Linear contrast	Quadratic contrast
Feed cost/fish	23.55 ± 0.0^e^	26.55 ± 0.0^d^	39.50 ± 0.70^c^	43.32 ± 0.95^b^	46.33 ± 0.74^a^	0.62	< 0.01	< 0.01
EFCR	3.13 ± 0.42^a^	2.48 ± 0.21^b^	2.29 ± 0.01^c^	2.22 ± 0.21^c^	1.99 ± 0.14^d^	0.41	< 0.01	< 0.01
Value of fish produced	63.19 ± 0.31^e^	71.49 ± 0.78^d^	107.19 ± 0.49^c^	126.11 ± 0.45^b^	140.17 ± 0.32^a^	0.50	< 0.01	0.01
Net profit	39.64 ± 0.32^e^	44.94 ± 0.77^d^	67.69 ± 0.22^c^	82.44 ± 1.39^b^	93.85 ± 0.42^a^	0.51	< 0.01	0.09
Net profit margin (%)	63.00 ± 0.0^b^	63.00 ± 0.0^b^	63.00 ± 0.01^b^	65.0 ± 0.01^a^	67.00 ± 0.01^a^	0.01	< 0.01	0.02
Profit index (PI)	2.56 ± 0.01^d^	2.67 ± 0.01^c^	2.76 ± 0.01^b^	2.76 ± 0.10^ab^	2.80 ± 0.01^a^	0.01	< 0.01	< 0.01
Incidence of cost (IC)	1.49 ± 0.01^a^	1.48 ± 0.02^a^	1.47 ± 0.02^a^	1.39 ± 0.04^b^	1.32 ± 0.02^b^	0.02	< 0.01	0.02
BCR	2.68 ± 0.01^a^	2.69 ± 0.03^a^	2.71 ± 0.04^b^	2.89 ± 0.07^b^	3.03 ± 0.04^b^	0.02	< 0.01	0.02
Gross revenue	63.19 ± 0.31^e^	71.49 ± 0.78^d^	107.19 ± 0.49^c^	126.11 ± 0.45^b^	140.17 ± 0.32^a^	0.50	< 0.01	0.01

Means that do not share a letter across the rows are significantly different (P< 0.05).

Because of this efficiency, the 121.8% expansion in the total market value of the harvested fish easily outpaced the higher manufacturing costs, generating a 136.8% increase in net profit per individual fish. This financial performance was statistically supported by the linear and quadratic trends observed for both the BCR and the PI across the treatments. These curves show that the higher costs of the PFE were fully offset at the 20.0 g/kg inclusion level, indicating where investing in this nutraceutical maximizes net returns.

## 4 Discussion

### 4.1 Bioactive profiling and fatty acid synergy

The complex volatile and phytochemical profile of PFE resolved by GC-MS establishes the baseline dynamics for the physiological and financial shifts observed in the feeding trials. Because the extract matrix is heavily weighted toward energy-dense LCFAs, its integration into aquafeeds directly changes how the diet is processed by the fish. The high baseline concentrations of linoleic acid (ω-6, 20.46%) and oleic acid (12.49%) are highly beneficial for Nile tilapia. Linoleic acid acts as the core dietary precursor for synthesizing long-chain polyunsaturated fatty acids (LC-PUFAs) and eicosanoids. These molecules serve as the primary structural components for cell membrane integrity and govern immune signalling pathways in Nile tilapia (Baker, 2018). At the same time, oleic acid is a major monounsaturated fatty acid (MUFA) that contributes direct lipid-clearing and antioxidant protection, shielding vulnerable cell walls from oxidative degradation (Allman-Farinelli et al., 2005; Islam et al., 2023). The substantial presence of myristic acid (14.53%) gives us a scientific clue that the PFE could also be used to stabilize gut health under intensive culture conditions, as this saturated fatty acid are known to exert direct antimicrobial control by disrupting the lipid bilayers of opportunistic bacterial cell membranes and blocking virulence pathways (Rani et al., 2022; Umma et al., 2025). Importantly, while raw West African botanicals are frequently held back by heavy anti-nutritional traits, the exceptionally low tannin content (0.26%) in this specific extract ensures that these valuable lipids are fully bioavailable. The fish can absorb them across the gut wall without suffering from intestinal inflammation or blocked protein pathways.

By establishing the precise chemical fingerprint of PFE, these data resolve a major problem in sub-Saharan aquaculture; which is the lack of standardized, empirical baselines for indigenous phytobiotics. For commercial feed manufacturers, these findings move prekese out of the realm of unquantified traditional remedies and reclassify it as a predictable, high-value functional lipid and health supplement suited for intensive production systems.

### 4.2 Growth performance

The growth and feed utilization patterns across the treatment groups are a direct biological reflection of the extract’s underlying chemistry. By using graded PFE treatments up to 20.0 g/kg, we achieved an exceptional growth response that outpaces many conventional phytogenic trials in tilapia literature, closely matching high-efficiency alternative inputs like mango kernel meal (Sharker et al., 2025a). The coupled linear and quadratic trends across SGR and FCR reveal that PFE stimulates multiple metabolic pathways simultaneously. This growth curve appears to be driven initially by a sensory response; the clear rise in feed consumption shows that the extract functions as an excellent flavour enhancer. This is directly tied to the volatile profile found in our GC-MS analysis, where long-chain fatty acids and aromatic complexes act as natural attractants that improve feed palatability.

Beyond making the feed taste better, the linear and quadratic rises in PER show that the active components in PFE change how nutrients are processed internally. The fish assimilated the 35% crude protein in the base diet far more efficiently as PFE levels rose. These trends align with established literature suggesting that plant secondary metabolites can stimulate endogenous digestive enzyme secretion (e.g., proteases, lipases) and upregulate nutrient transport pathways across the intestinal brush border membrane (Li and Liu, 2025). However, as digestive enzyme activities and brush border transport kinetics were not directly quantified in the present study, these pathways should be viewed as plausible contributing mechanisms that warrant direct enzymatic and histological validation in future trials.

Our regression models give us an exact look at these physiological boundaries. While the FCR curve shows continuous efficiency gains across all treatments, the SGR vertex model reveals that the absolute biological peak rests at 29.86 g/kg. This threshold behaviour explains why growth accelerates smoothly up to 20.0 g/kg without hitting a premature plateau. It confirms that at the 20.0 g/kg level, the fish easily process the minor dietary tannin fraction (0.26%) without encountering the gut irritation or anti-nutritional blockages common with raw, unrefined plant meals (Gemede and Ratta, 2018). Hence, for the aquaculture industry, these curves provide a practical template for lowering feed costs. By using PFE at 20.0 g/kg, feed mills can drop biological FCR from 1.52 to 1.10. This allows intensive farms to generate substantially more fish biomass from less total feed, directly supporting the cost-effective and sustainable intensification of regional aquaculture.

### 4.3 Haematological and immune dynamics

The blood and immune profiles across the treatments demonstrate that PFE exerts a safe, highly regulatory influence on both red blood cell production and white blood cell behaviour. The linear increase in total WBCs and haematocrit values shows a highly active hematopoietic response. This fortified cellular profile provides the oxygen transport and immune baseline needed to sustain the rapid, 50.5% weight gain observed at the highest inclusion level (Hrubec et al., 2000). Because haemoglobin and PCV remained within perfectly healthy ranges without sudden drops, we can confirm that these PFE treatments do not cause haemolytic anaemia, proving the safety of the extract across the entire tested range.

The shifts observed in differential leucocyte proportions reveal subtle modulations in peripheral immune cells. While circulating lymphocyte proportions decreased to 25.50% in the 20.0 g/kg group, evaluation of the broader leucocyte profile indicates a corresponding rise in granulocytic lineages, such as eosinophils. This trend suggests a potential leukocyte redistribution in response to dietary bioactives like myristic and oleic acids. However, because functional immune responses (such as lysozyme activity, respiratory burst, complement pathways, or cytokine gene expression) were not directly measured, these haematological shifts reflect general systemic health improvements rather than definitive modulation of specific innate or adaptive immune cascades.

The sudden appearance of monocytes only in the mid-range 10.0 and 15.0 g/kg groups highlights a clear, dose-dependent operational window. Within these intermediate treatments, the plant metabolites safely spark macrophage differentiation. As the PFE dose reaches the 20.0 g/kg maximum, these cells likely leave the bloodstream and migrate into peripheral tissues to fortify mucosal barriers like the gut lining. This localized migration perfectly explains why they drop below detectable levels in circulating blood samples at the highest dose.

These cellular changes directly solve the scientific problem noted in our introduction: the lack of clear data on how local phytobiotics change the internal health and safety metrics of cultured fish. For commercial operators, these findings have immediate practical value. Intensive fish farming puts animals under constant stress from handling and crowding, leaving them vulnerable to disease. Our data prove that using PFE up to 20.0 g/kg gives feed manufacturers a natural way to strengthen the innate immune system of Nile tilapia (Harikrishnan et al., 2011). By building a better internal defence line, farmers can shield their stocks from seasonal disease outbreaks without using expensive synthetic antibiotics, creating a more stable and resilient farm ecosystem.

### 4.4 Hepatic metabolism and the antioxidant sparing effect

The serum chemistry and antioxidant shifts reflect a series of deep protective and metabolic adaptations driven by the extract’s components. The gradual increase in serum AST (peaking at 70.50 IU/L at the 20.0 g/kg level), ALT, ALP, and GGT might normally suggest liver stress or cell damage. However, because these changes occurred alongside a massive 50.5% boost in final weight, This suggests that the observed shifts may reflect up-regulated amino acid transamination and intermediate protein catabolism to support rapid somatic tissue building (Anderson and Siwicki, 1995). Nonetheless, in the absence of liver histology or liver-specific metabolic gene expression data, accelerated hepatic transamination remains a proposed metabolic explanation rather than a proven conclusion.

Importantly, our recorded enzyme values sit well below the toxic thresholds known for healthy Nile tilapia, where ALT reaches 45.25 IU/L (Sharker et al., 2024) and safe limits for AST reach 104 IU/L and ALT up to 25 IU/L without causing structural damage (Sharker et al., 2026). This broad safety margin confirms that our dietary treatments accelerated metabolic efficiency without causing tissue leakage. Similarly, elevated GGT levels at 15.0 and 20.0 g/kg thresholds may point to an adaptive hepatic response involved in processing extract-derived phytochemicals (Hrubec et al., 2000), though definitive evidence of specific liver detoxification pathways requires direct molecular or histopathological verification.

Regarding oxidative balance, the observed declines in endogenous antioxidant enzyme activities (SOD and CAT), alongside stable GSH and lower MDA levels at 15.0 g/kg, suggest a potential exogenous radical-scavenging contribution from PFE’s polyphenols and flavonoids. Exogenous antioxidants may directly neutralize reactive species, potentially reducing the metabolic demand for endogenous enzyme synthesis. This possibly explains the excellent 1.10 biological FCR and enhanced protein efficiency seen in the high-inclusion groups. However, because reactive oxygen species (ROS) production, total antioxidant capacity (TAC), and direct oxidative damage pathways were not explicitly measured, this antioxidant-sparing mechanism remains a plausible hypothesis rather than an established fact.

The nitrogen processing across these treatments also provides clear evidence of tissue preservation. Linear increases in serum urea suggest active amino acid turnover aligned with accelerated growth (Hyodo et al., 2014), while significant reductions in serum creatinine at 10.0 – 20.0 g/kg reflect normal renal clearance and nitrogenous waste processing (Abdel-Tawwab and Abbass, 2017). While lower creatinine levels are consistent with healthy protein metabolism and renal clearance, direct claims regarding muscle tissue preservation or protein turnover cannot be definitively concluded without muscle histology, body composition, or protein turnover assays This metabolic stabilization matches our positive PER growth data, and also suggest that PFE channels dietary protein away from metabolic waste and directly into edible biomass.

These clear metabolic insights fix the core issue previously raised on the lack of functional data on how West African phytobiotics regulate tissue kinetics in cultured fish. For the industry, this offers a valuable operational tool. Managing metabolic stress and cell oxidation is a constant, expensive challenge in intensive fish farming. Our results show that using PFE up to 20.0 g/kg gives mill operators a reliable, natural tool to protect fish from oxidative stress while accelerating their protein absorption. By optimizing internal energy use and protecting muscle tissue, this feeding strategy ensures that intensive farms can extract maximum growth from every kilogram of feed, providing excellent production security and highly predictable financial returns.

### 4.5 Economic viability and value-added aquaculture

The financial metrics from these treatments challenge the common assumption in aquaculture that cheaper feed formulations lead to higher profits. The control diet (0.0 g/kg, PFE), though the cheapest to manufacture, produced the worst EFCR (3.13) and the lower net returns. In contrast, while grading PFE levels up to 20.0 g/kg elevated feed costs quadratically by 96.7%, this upfront investment was fully recovered through the biological performance of the fish.

The scientific explanation for this economic resilience lies in the metabolic and tissue-preservation improvements observed in the preceding data sections. The dense supply of LCFAs (like linoleic acid) and the antioxidant sparing effect of the polyphenols in PFE allowed the 20.0 g/kg group to extract far more nutritional value from each kilogram of feed. Because the fish broke down and retained the 35% crude protein base diet more efficiently, the EFCR fell to 1.99, matching high-efficiency patterns recorded for other functional crop byproducts in tropical aquaculture (Sharker et al., 2025b). This biological efficiency reduced the incidence of cost (IC), meaning far less money was lost to unabsorbed feed dissolving into the pond bottom.

Thanks to this improved nutrient absorption, the 121.8% jump in total biomass value absorbed the higher formulation expenses. The strong linear and quadratic upward trends in both BCR and PI confirm this relationship statistically, proving that the financial returns follow a highly predictable trend line. The 20.0 g/kg treatment represents a state of high allocative efficiency, where the expense of the plant extract is entirely validated by the 136.8% increase in net profit per fish.

These financial patterns resolve the previous issue raised on the lack of clear microeconomic profiling for local phytobiotics, which has caused many farmers to hesitate to use them. For the commercial sector, these figures establish an essential financial baseline. Feed represents 60 – 70% of total running costs on intensive fish farms. By proving that a 20.0 g/kg inclusion of PFE lowers EFCR and expands net margins, we offer commercial feed mills and local tilapia farmers a practical method to maximize their returns. Shortening production timelines through faster growth while protecting net margins directly supports United Nations SDG-2 (Zero Hunger) by reducing financial risks, increasing local food supply, and improving the economic livelihoods of fish farmers across sub-Saharan Africa.

## 5 Conclusion

In conclusion, this study establishes *Tetrapleura tetraptera* fruit extract (PFE) as a highly effective functional additive for Nile tilapia (*Oreochromis niloticus*) diets. At the 20.0 g/kg inclusion threshold, the extract significantly optimizes growth trajectories and nutrient assimilation through a combination of appetite stimulation, elevated protein efficiency, and a clear exogenous antioxidant-sparing mechanism. While the plant metabolites accelerate liver metabolic pathways, serum biomarkers confirm these shifts reflect healthy protein synthesis to support rapid muscle deposition rather than tissue damage. Economically, the initial manufacturing cost penalty of PFE is fully recovered through a major drop in biological and economic feed conversion ratios, ultimately yielding a 136.8% increase in net profit per fish and offering a practical framework for the sustainable intensification of tropical aquaculture.

To successfully transition this phytobiotic into commercial feed mills, future research must move beyond static aquarium trials and address real-world operational bottlenecks. First, field-scale validation trials in open earthen ponds and commercial cage systems are required to confirm that these feed efficiencies hold true under natural environmental and microbial variations. Second, future studies should evaluate the protective capacity of dietary PFE under acute production stressors, testing whether its antioxidant properties can suppress systemic cortisol spikes and lipid peroxidation during handling, high-density crowding, and hypoxia challenges. Finally, given the extract’s rich fatty acid profile, it is critical to investigate downstream effects on the final product by profiling fillet lipid deposition, measuring oxidative shelf-life during cold storage, and conducting sensory panels to ensure consumer marketability.

## Data Availability

The raw data supporting the conclusions of this article will be made available by the authors, without undue reservation.
